# Electrical risk score as a predictor of acute ischemic stroke and 30-day mortality: a prospective observational study

**DOI:** 10.1186/s12883-026-04835-3

**Published:** 2026-03-19

**Authors:** Metin Ocak, Pinar Henden Cam, Mustafa Begenc Tascanov, Metin Yadigaroglu, Yusuf Koksal, Ulkuhan Duzgun

**Affiliations:** 1 Faculty of Medicine, Department of Emergency Medicine, Samsun University, Kadıköy, Barış Blv. No:199, İlkadım/Samsun, 55090 Türkiye; 2 Samsun Gazi State Hospital, Emergency Medicine Clinic, Samsun, Türkiye; 3 Faculty of Medicine, Department of Cardiology, Samsun University, Samsun, Türkiye; 4 Primary Health Care Corporation, Doha, Qatar; 5 Gulhane Faculty of Medicine, Department of Neurology, University of Health Sciences, Ankara, Türkiye

**Keywords:** Ischemic Stroke, Electrical Risk Score, Electrocardiogram, Prognosis, Emergency Medicine

## Abstract

**Background:**

Ischemic stroke (IS) remains a leading cause of death and disability worldwide. Stroke–heart syndrome, triggered by acute cerebral ischemia, causes autonomic dysfunction that can result in myocardial injury, arrhythmias, and sudden cardiac death, even in the absence of preexisting cardiac disease. The Electrical Risk Score (ERS), derived from standard electrocardiographic (ECG) parameters, has prognostic value in cardiac conditions, but its utility in IS populations remains unclear. This study aimed to evaluate the discriminative performance of ERS between confirmed acute IS patients and matched healthy controls, and to assess its prognostic value for 30-day all-cause mortality among patients with IS.

**Methods:**

This prospective observational study enrolled 200 patients with acute IS and 101 age- and sex-matched healthy controls at a tertiary emergency department. ERS (range: 0–6) was calculated using six predefined ECG parameters: heart rate, QTc interval, Tp-e interval, frontal QRS-T angle, QRS transition zone, and left ventricular hypertrophy.

**Results:**

ERS was significantly higher in IS patients compared to controls (*p* < 0.001). ERS independently predicted 30-day mortality (adjusted OR 1.66, 95% CI 1.14–2.42; *p* = 0.008). Receiver operating characteristic (ROC) analysis showed that ERS ≥ 3 discriminated confirmed IS cases from matched controls with 61% sensitivity and 77% specificity (AUC: 0.725; 95% CI: 0.669–0.781; PPV: 0.841; NPV: 0.500; *p* < 0.001). ERS ≥ 4 predicted 30-day mortality with 55% sensitivity and 74% specificity (AUC: 0.681; 95% CI: 0.585–0.778; PPV: 0.267; NPV: 0.907; *p* = 0.002). Higher ERS values showed a significant positive correlation with stroke severity based on Glasgow Coma Scale (GCS) scores (*p* = 0.005).

**Conclusion:**

ERS is a simple bedside ECG-based tool with moderate accuracy for predicting acute IS and short-term mortality. It may support early risk stratification in settings with limited stroke-specific resources. Further multicenter studies are needed for validation.

## Introduction

Ischemic stroke (IS) is a leading cause of death and long-term disability worldwide. Its global burden continues to grow due to ageing populations and persistent cardiovascular risk factors [1]. Although significant advances have been made in acute stroke management, the early identification of patients at increased risk for neurological deterioration or short-term mortality continues to represent a critical gap in emergency care [2].

The connection between the brain and heart, often called stroke–heart syndrome, has gained increasing attention in recent years. This condition includes a range of heart problems such as arrhythmias, repolarisation abnormalities, myocardial dysfunction, and sometimes sudden cardiac death, even in patients without known heart disease [3–5]. These complications are associated with poorer functional outcomes and elevated mortality risk. The underlying pathophysiology is primarily attributed to autonomic nervous system dysregulation and catecholamine surges triggered by cerebral injury, particularly when the insular cortex is affected [6–9]. This autonomic imbalance may promote myocardial inflammation and arrhythmogenesis, further exacerbating cardiovascular instability in the acute phase of stroke.

Standard 12-lead electrocardiography (ECG) is a widely accessible, low-cost bedside tool routinely employed in the emergency department (ED) to assess cardiac risk. Although parameters such as the corrected QT interval (QTc), the T peak–to–T end (Tp-e) interval, and the frontal QRS-T angle have been investigated individually for prognostic value in acute IS, their clinical utility remains limited when used in isolation. These markers are frequently influenced by confounding factors, including electrolyte disturbances, pharmacologic agents, and baseline cardiovascular conditions, which may compromise their specificity and predictive accuracy [10–12].

To overcome the limitations of individual ECG parameters, the Electrical Risk Score (ERS) was introduced as a composite ECG-derived metric that integrates six variables: heart rate, QTc interval, Tp-e interval, QRS-T angle, QRS transition zone, and evidence of left ventricular hypertrophy (LVH). ERS has demonstrated prognostic value in cardiac populations, where it has been associated with an increased risk of sudden cardiac death and major adverse cardiovascular events [13–15]. However, its clinical utility in the setting of IS has not yet been systematically evaluated.

In this study, we sought to evaluate the discriminative performance of ERS between confirmed IS patients and matched controls, as well as its ability to predict 30-day all-cause mortality in patients with IS. We hypothesise that elevated ERS values will correlate with greater clinical severity and higher short-term mortality risk, thereby offering a practical and reproducible triage tool in emergency settings where immediate access to advanced neuroimaging or standardised neurologic scoring systems may be limited.

## Materials and methods

### Study design and setting

This prospective, observational, single-centre study was conducted in the ED of Samsun University Training and Research Hospital, a tertiary care academic institution in northern Türkiye. The ED manages over 100,000 visits annually and serves as a regional referral centre for time-sensitive emergencies, including acute cerebrovascular events. The study period spanned from November 1, 2022, to October 31, 2023.

The study protocol was approved by the Samsun University Clinical Research Ethics Committee (Approval No: SÜKAEK/2022/9/3, dated October 5, 2022), and all procedures were conducted in accordance with the Declaration of Helsinki. Written informed consent was obtained from all participants or their legal representatives prior to enrollment.

### Participants

#### Inclusion and exclusion criteria

Eligible participants were adults (≥ 18 years) who presented to the ED with clinical signs suggestive of acute IS, subsequently confirmed by neuroimaging—either non-contrast cranial computed tomography (CT) and/or diffusion-weighted magnetic resonance imaging (MRI)—as interpreted by board-certified radiologists.

Exclusion criteria were:

- Age < 18 years.
- Hemorrhagic stroke or stroke mimics.
- IS within the past 6 months.
- History of significant structural cardiac disease (e.g., moderate-to-severe valvular disease or cardiomyopathy).
- Documented arrhythmias, recent myocardial infarction (< 3 months), or presence of a pacemaker.
- Second- or third-degree atrioventricular block or complete bundle branch block.
- Terminal illness or active malignancy.
- Refusal or inability to provide informed consent.
- Inter-hospital transfer during the acute phase.
- Missing 30-day outcome data.

#### Control group

**Fig. 1:**
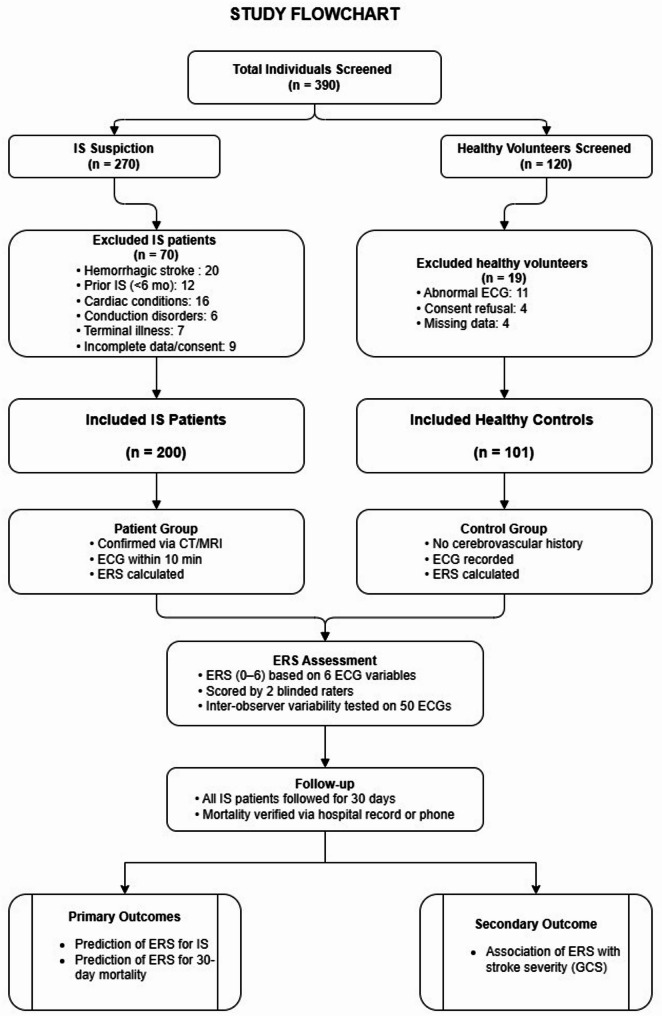
Study Flowchart of participant enrollment, assessment, and outcomes

The control group consisted of 101 age- and sex-matched healthy volunteers recruited during concurrent general health screenings at the hospital. Participants had no history or clinical symptoms suggestive of cerebrovascular disease. Control subjects underwent the same baseline assessments, including 12-lead ECG and laboratory testing (See Fig. 1: Study Flowchart). All control data were obtained solely for research purposes, following ethics committee approval and informed consent.

### Data collection

Sociodemographic variables (age, sex), comorbid conditions (e.g., hypertension, diabetes mellitus, coronary artery disease, smoking), and vital signs (systolic/diastolic blood pressure, heart rate, respiratory rate, body temperature, and oxygen saturation) were recorded upon ED admission.

IS severity was evaluated using the Glasgow Coma Scale (GCS), as assessed by an emergency physician trained in neurological evaluation [16]. Laboratory parameters included complete blood count, serum glucose, creatinine, electrolytes, C-reactive protein (CRP), cardiac troponin-I, CK-MB, and D-dimer. All data were collected prospectively using standardised case report forms and independently validated by a third investigator to ensure data quality.

Mortality data were obtained from hospital records and verified through telephone follow-up when necessary.

### ERS Assessment

All participants underwent a 12-lead ECG within 10 min of ED presentation, using standardised acquisition settings (25 mm/s speed, 10 mm/mV amplitude, supine position). ERS was calculated by assigning one point for each of the following six predefined ECG abnormalities:

1. Heart rate > 75 bpm.
2. QTc > 450 ms in men or > 460 ms in women.
3. Tp-e interval > 89 ms.
4. Evidence of LVH per Sokolow–Lyon criteria.
5. QRS transition zone at V4 or later.
6. Frontal QRS-T angle > 90°.

The Tp-e interval was measured manually using callipers and magnification on printed ECG tracings, from the peak to the end of the T wave in the lead with the clearest morphology. LVH was determined according to the Sokolow–Lyon criteria, which included the presence of any of the following four parameters:

- The sum of the S wave in V1 and the largest R wave in V5 or V6 > 35 mm.
- R wave amplitude > 25 mm in V5 or V6.
- The sum of the R wave in lead I and the S wave in lead III > 25 mm.
- R wave amplitude > 13 mm in aVL or > 20 mm in aVF.

Patients who met at least one of these criteria were classified as having LVH and received 1 point in the ERS calculation [13, 17]. ERS ranged from 0 to 6. Two independent physicians, a board-certified emergency physician and a cardiologist, assessed all ECGs while blinded to clinical outcomes. Discrepancies were resolved by consensus.

To evaluate inter- and intra-observer variability, 50 ECGs were randomly selected for repeated measurement of QTc and Tp-e intervals. The coefficient of variation (CV) was calculated for both parameters. Inter-observer CVs for QTc and Tp-e intervals were 2.1% and 2.7%, respectively, indicating excellent measurement consistency.

### Study outcomes

#### Primary outcomes

- To evaluate the discriminative performance of ERS between confirmed IS patients and matched healthy controls.
- To assess the predictive value of ERS for 30-day all-cause mortality among patients with IS.

#### Secondary outcome

- To examine the association between ERS and IS severity at the time of admission, categorised according to the GCS as mild (GCS 14–15), moderate (GCS 9–13), and severe (GCS 3–8) [16].

### Statistical analysis

Sample size was calculated using G*Power (version 3.1) based on a moderate effect size (Cohen’s d = 0.5), an alpha of 0.05, and power (1–β) of 0.80, following the recommended methods for clinical research sample estimation [18]. The minimum required sample was 176 participants. To improve robustness and account for potential data loss, 200 IS patients and 101 control subjects were enrolled.

Statistical analyses were performed using IBM SPSS Statistics, version 20.0 (IBM Corp., Armonk, NY). Normality of continuous variables was assessed using the Kolmogorov–Smirnov test. Data were expressed as mean ± standard deviation (SD) or median with interquartile range (IQR), depending on distribution. Categorical variables were presented as frequencies and percentages.

Between-group comparisons were conducted using the Student’s t-test or Mann–Whitney U test for continuous variables, and the chi-square test or Fisher’s exact test for categorical variables. Univariate logistic regression was used to explore associations between ERS and study outcomes. Variables with *p* < 0.10 in the univariate analysis and with known clinical relevance were included in multivariate models to balance robustness and parsimony. Adjusted odds ratios (OR) with 95% confidence intervals (CI) were reported. Mortality analysis was restricted to the IS group, comparing survivors and non-survivors within this cohort. For mortality analysis, 30-day death was coded as 1 and survival as 0 in the logistic regression models.

Receiver operating characteristic (ROC) analysis was used to evaluate the discriminative ability of ERS in predicting IS and 30-day mortality. Although optimal cut-off values were initially identified using Youden’s index, clinically applicable integer thresholds were reported to enhance bedside interpretability. The area under the curve (AUC), sensitivity, specificity, and optimal thresholds were reported for each outcome. All tests were two-tailed, with p-values < 0.05 considered statistically significant.

## Results

### Participant characteristics

Baseline demographic and clinical characteristics of the IS group (*n* = 200) and the control group (*n* = 101) are summarised in Table 1. The mean age was similar between groups (74.78 ± 11.60 vs. 74.15 ± 11.01 years; *p* = 0.573). No significant differences were observed in systolic blood pressure (SBP), diastolic blood pressure (DBP), mean arterial pressure (MAP), body temperature, oxygen saturation, or most laboratory parameters, including troponin, D-dimer, and others (*p* > 0.05). However, white blood cell (WBC) count (9.11 ± 3.61 vs. 8.24 ± 2.52 × 10³/µL; *p* = 0.026) and neutrophil count (5.70 [4.21–7.59] vs. 4.76 [3.86–6.78] ×10³/µL; *p* = 0.023) were significantly higher in the IS group. There were no significant differences in gender distribution, smoking status, or the prevalence of diabetes mellitus, hypertension, coronary artery disease, chronic obstructive pulmonary disease, or congestive heart failure between the groups (*p* > 0.05) (Table 1).

**Table 1:** Baseline characteristics of patients with IS and the control group

Variables (mean±SD)	Patient Group (*n* = 200)	Control Group (*n* = 101)	*p*-values
Age, year	74.78±11.60	74.15±11.01	0.573
SBP (mmHg)	146.58±25.45	144.33±25.40	0.478
DBP (mmHg)	84.13±15.89	82.27±13.55	0.586
MAP (mmHg)	104.29±18.52	102.14±17.34	0.527
Body temperature (°C)	36.21±2.04	36.25±2.04	0.373
Saturation (%)	96.25±1.84	96.53±1.68	0.152
WBC (x103/µL)	9.11±3.61	8.24±2.52	0.026
Haemoglobin (g/dL)	12.98±1.84	12.69±1.48	0.188
Thrombocyte (x10³/µL)	245 (193–292)	233(184–293)	0.577
Neutrophil (x10³/µL)	5.70 (4.21–7.59)	4.76(3.86–6.78)	0.023
Lymphocyte (x10³/µL)	1.73 (1.24–2.48)	1.78 (1.28–2.47)	0.876
Sodium (mmol/L)	136.91±4.82	136.68±3.69	0.953
Potassium (mmol/L)	4.08±0.58	4.08±0.50	0.949
Urea (mg/dL)	41 (31–54)	40 (31–53)	0.745
Creatinine (mg/dL)	1.08±0.58	1.07±0.65	0.528
Glucose (mg/dL)	161.51±88.09	146.73±70.38	0.144
CRP (mg/L)	19.00 ±49.31	16.14±52.60	0.209
CK-MB (ng/mL)	3.23±2.37	3.32±1.86	0.263
Troponin (ng/mL)	0.08±0.20	0.04±0.08	0.061
D-Dimer (µg/L)	0.79±0.72	0.67±0.60	0.210
Sex			
Female	119 (59.5)	63 (62.4)	0.630
Male	81 (40.5)	38 (37.6)	
Smoking	60 (30)	25 (24.8)	0.340
DM	80 (40)	40 (39.6)	0.947
HT	168 (84)	85 (84.2)	0.972
COPD	4 (2)	1 (1)	0.517
CAD	73 (36.5)	26 (25.7)	0.061
CHF	26 (13)	11(10.9)	0.599

*IS* Ischemic stroke,* SBP *Systolic blood pressure* DBP *Diastolic blood pressure* MAP *Mean arterial pressure,* WBC *White blood cell count* CRP*C-reactive protein,* CK-MB *Creatine kinase–myocardial band,* DM *Diabetes mellitus,* HT *Hypertension,* COPD *Chronic obstructive pulmonary disease,* CAD *Coronary artery disease,* CHF *Congestive heart failure

### ECG parameters and ERS

Electrocardiographic assessment revealed significantly higher heart rates (82.8 ± 19.9 vs. 75.3 ± 18.6 bpm, *p* < 0.001) and ERS (2.82 ± 1.35 vs. 1.79 ± 0.88, *p* < 0.001) in the IS group compared to controls. No statistically significant differences were noted in QTc, Tp-e interval, and frontal QRS-T angle between groups (Table 2).

**Table 2:** Comparison of ECG-derived parameters and ERS between patients with IS and controls

Variable	Stroke Group (*n* = 200)	Control Group (*n* = 101)	*p*-value
Pulse (bpm)	82.8 ± 19.9	75.3 ± 18.6	< 0.001
Frontal QRS-T angle (°)	40 (14–95)	29 (13–71)	0.055
QTc interval (ms)	418 (403–433)	420 (404–432)	0.906
Tp-e interval (ms)	73.1 ± 44.9	63.3 ± 37.3	0.099
ERS (0–6)	2.82 ± 1.35	1.79 ± 0.88	< 0.001*

*IS*Ischemic stroke,* QTc *corrected QT interval,* Tp-e *Tpeak-Tend interval,* ERS *Electrical Risk Score,* bpm*beats per minute

*Continuous variables are presented as mean ± SD or median (IQR), as appropriate

### Primary outcome 1: discriminative performance of ers between is patients and controls

In univariate analysis, WBC and ERS were significantly associated with IS diagnosis (*p* < 0.05). However, in multivariate logistic regression analysis, only ERS remained an independent predictor (OR: 2.091, 95% CI: 1.627–2.688, *p* < 0.001), whereas WBC lost statistical significance (OR: 1.026, 95% CI: 0.931–1.132, *p* = 0.601) (Table 3).

**Table 3:** Logistic regression analysis of predictors for IS diagnosis

Variable	Univariate OR (95% CI)	*p*-value	Multivariate OR (95% CI)	*p*-value (multi)
WBC	1.01 (1.002–1.018)	0.034	1.026 (0.931–1.132)	0.601
ERS	2.056 (1.626–2.599)	< 0.001	2.091 (1.627–2.688)	< 0.001
Age	1.005 (0.984–1.026)	0.655		
Smoking	1.303 (0.756–2.244)	0.340		
Hypertension	0.988 (0.514–1.901)	0.972		
CRP	1.001(0.996–1.006)	0.642		

*IS *Ischemic stroke,* WBC *White blood cell count,* ERS *Electrical Risk Score,* CRP*C-reactive protein,*OR *Odds ratio,*CI *Confidence interval

### Primary outcome 2: ers as a predictor of 30-day mortality

Among IS patients, 29 deaths occurred within 30 days. In univariate analysis, age, CRP, and ERS were significantly associated with 30-day mortality (*p* < 0.05). In multivariate analysis, all three variables remained independent predictors: age (OR: 1.063, 95% CI: 1.014–1.113, *p* = 0.011), CRP (OR: 1.012, 95% CI: 1.004–1.019, *p* = 0.002), and ERS (OR: 1.662, 95% CI: 1.142–2.419, *p* = 0.008) (Table 4).

**Table 4:** Logistic regression analysis of predictors for 30-day mortality in patients with IS

Variable	Univariate OR (95% CI)	*p*-value	Multivariate OR (95% CI)	*p*-value
Age	1.082 (1.035–1.131)	< 0.001	1.063 (1.014–1.113)	0.011
CRP	1.016 (1.008–1.024)	< 0.001	1.012 (1.004–1.019)	0.002
ERS	1.643 (1.195–2.259)	0.002	1.662 (1.142–2.419)	0.008
Hypertension	2.872 (0.648–12.737)	0.165		

*IS *Ischemic stroke,* CRP *C-reactive protein,* ERS *Electrical Risk Score,* OR *Odds ratio,* CI *Confidence interval

### Secondary outcome: association between ERS and IS severity

When stratified by IS severity according to GCS categories, a stepwise increase in ERS values was observed with worsening neurological status. One-way ANOVA revealed a significant difference across GCS strata (*p* = 0.002), with post hoc analysis confirming a statistically significant difference between the mild (GCS 14–15) and severe (GCS 3–8) groups (*p* = 0.005).

### ROC curve analysis

**Fig. 2:**
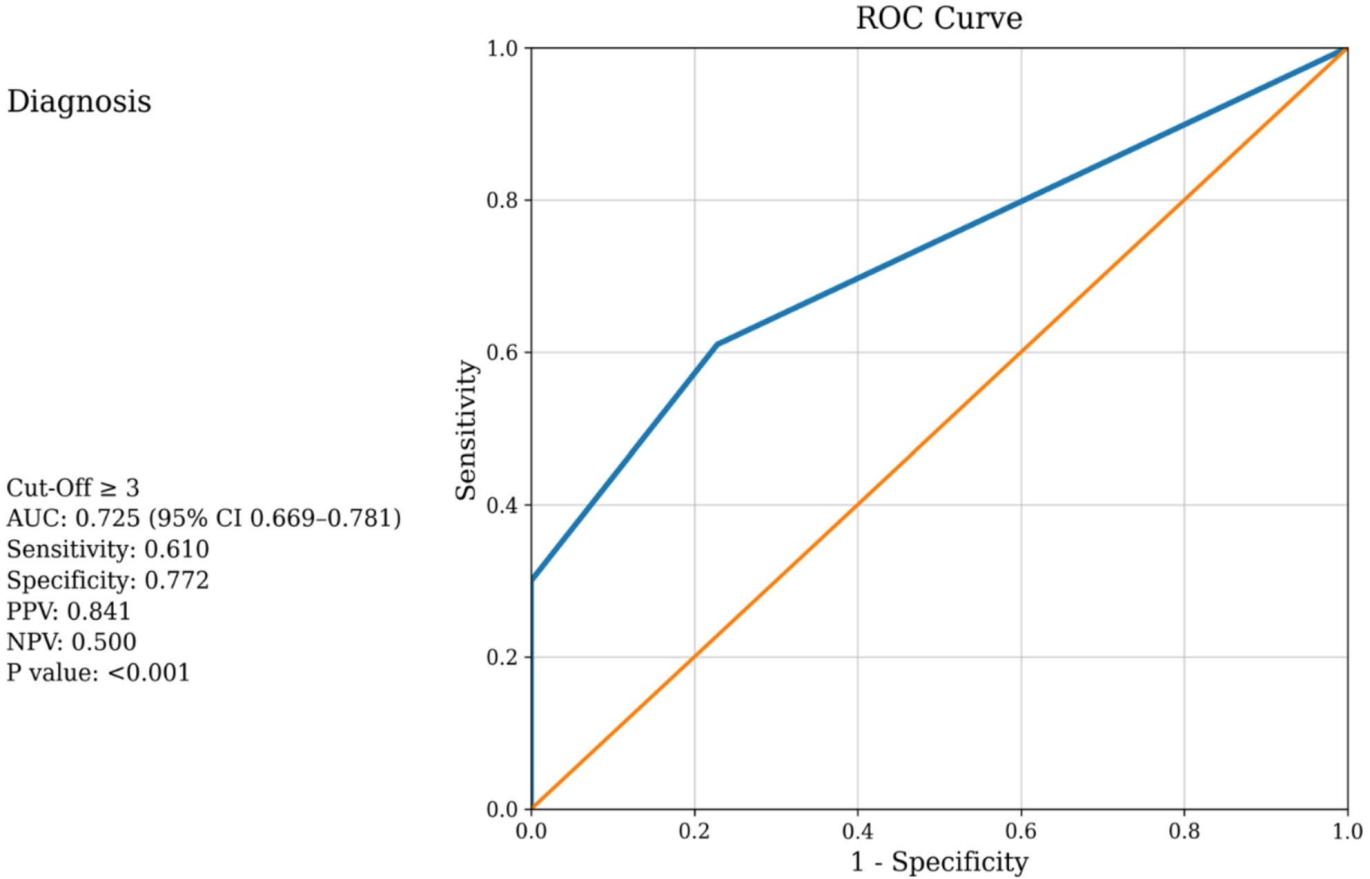
ROC curve of the Electrical Risk Score for the prediction of IS

**Fig. 3:**
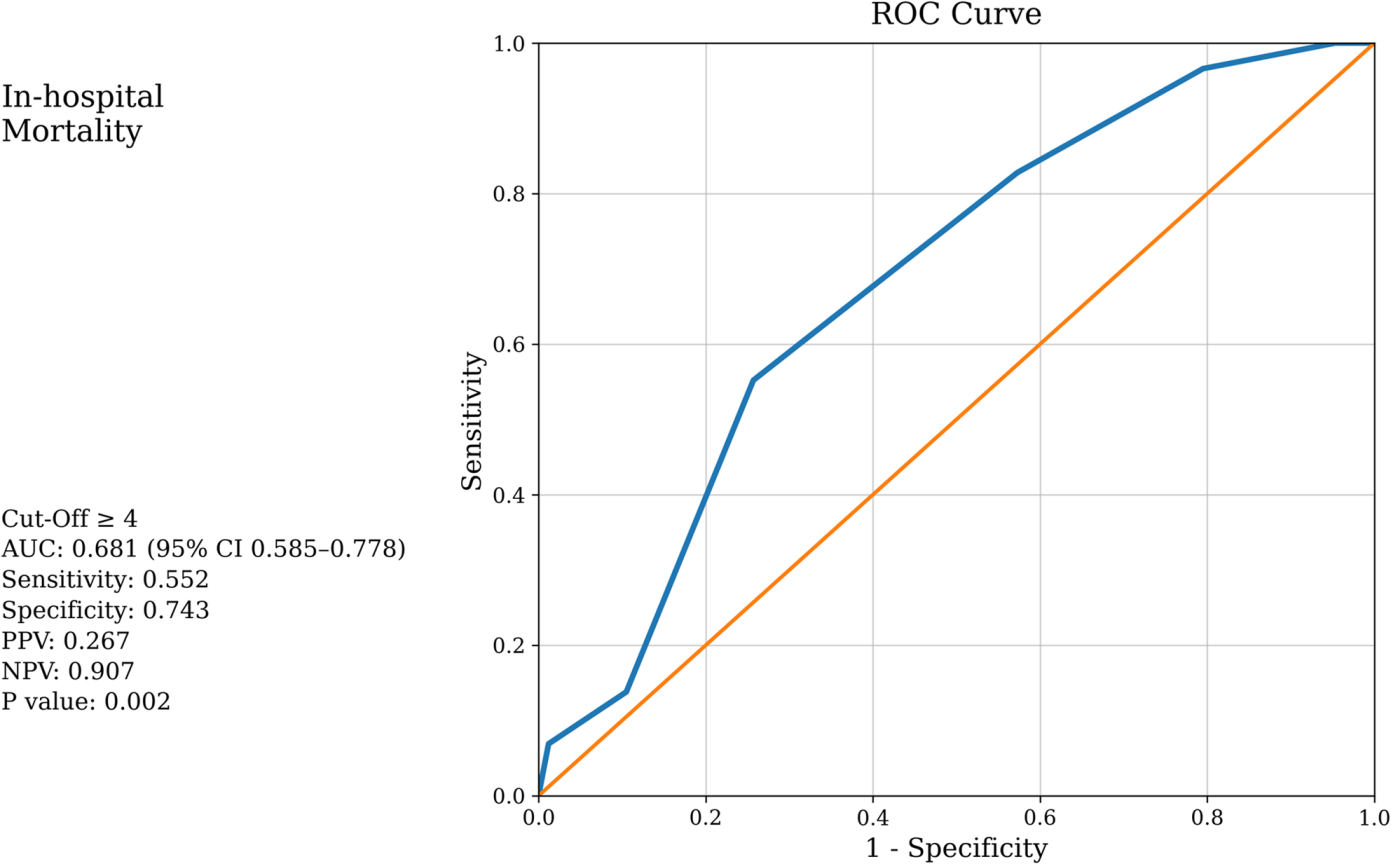
ROC curve of the Electrical Risk Score for predicting 30-day mortality in patients with IS

ROC analysis demonstrated that an integer ERS threshold of ≥ 3 discriminated confirmed IS cases from matched controls with 61% sensitivity and 77% specificity (AUC = 0.725; 95% CI: 0.669–0.781; Positive predictive value (PPV): 0.841; Negative predictive value (NPV): 0.500; *p* < 0.001) (Fig. 2). For predicting 30-day mortality, an ERS threshold of ≥ 4 yielded 55% sensitivity and 74% specificity (AUC = 0.681; 95% CI: 0.585–0.778; PPV: 0.267; NPV: 0.907; *p* = 0.002) (Fig. 3). Although optimal cut-off values derived from Youden’s index were fractional (e.g., 3.5), integer thresholds were reported to enhance clinical applicability and bedside interpretability.

### ERS distribution and mortality risk

**Fig. 4:**
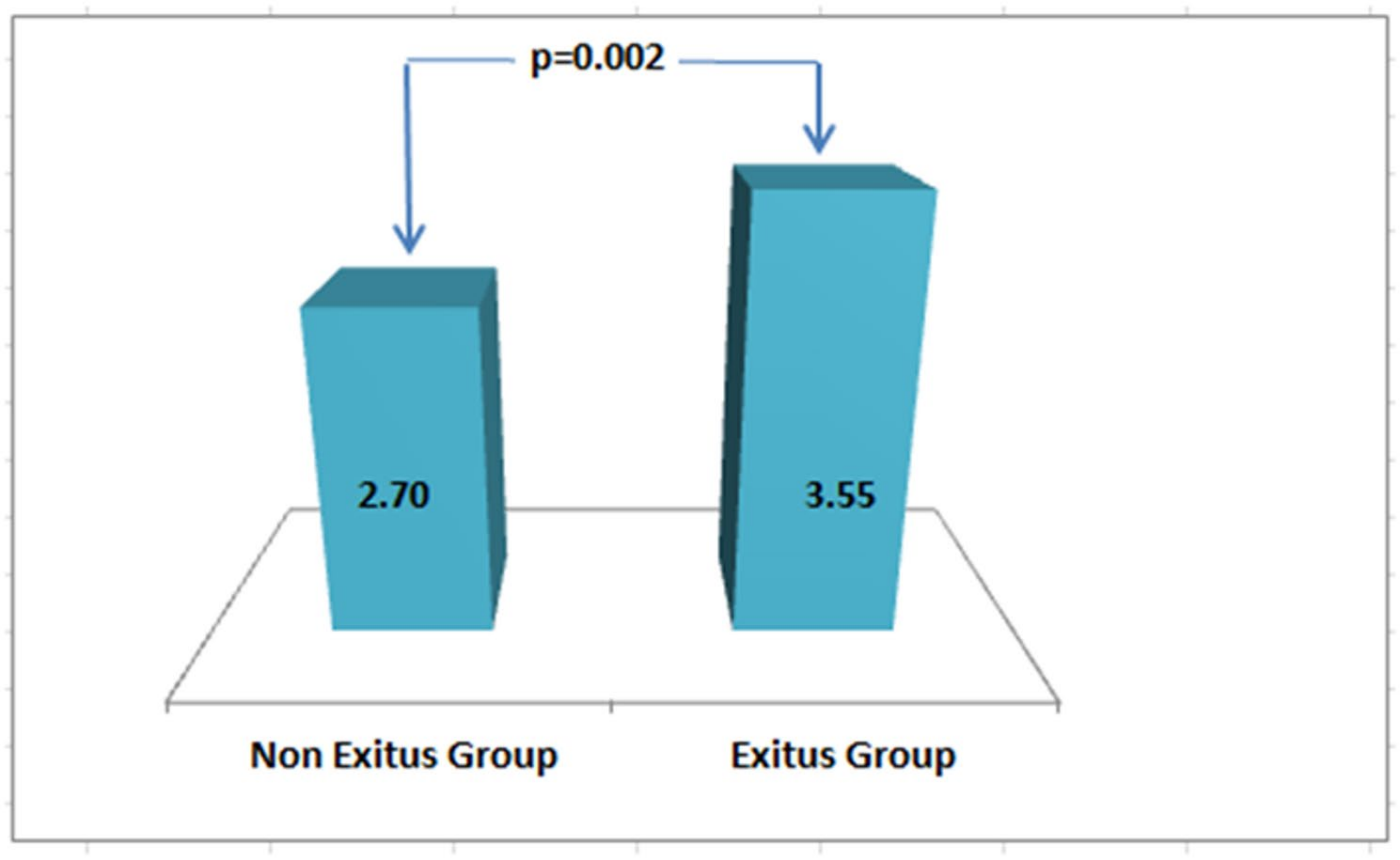
Distribution of the ERS according to 30-day mortality status in patients with IS

When participants were grouped according to the clinically applicable ERS threshold (< 4 vs. ≥ 4), 24 of the 29 deaths (82.8%) occurred in the ERS ≥ 4 group (*p* = 0.009). Furthermore, the mean ERS was significantly higher in deceased patients (3.55 ± 1.2) compared to survivors (2.70 ± 1.3) (*p* = 0.002), supporting the utility of ERS in early mortality risk stratification (Fig. 4).

### Correlation analysis

Spearman correlation analysis revealed weak but statistically significant positive correlations between ERS and WBC count (*r* = 0.294, *p* < 0.001), haemoglobin level (*r* = 0.173, *p* = 0.003), age (*r* = 0.149, *p* = 0.010), and IS severity as measured by GCS (*r* = 0.252, *p* < 0.001). No significant correlation was observed between ERS and CRP, serum sodium, potassium levels, or blood pressure (Table 5).

**Table 5:** Correlation between ERS and clinical/laboratory parameters

Parameter	Correlation Coefficient (*r*)	*p*-value
Age	0.149	0.010*
SBP	0.003	0.963
DBP	0.012	0.838
Hemoglobin	0.173	0.003*
WBC	0.294	< 0.001*
CRP	0.111	0.055
Potassium	-0.002	0.978
Sodium	-0.035	0.541
GCS severity	0.252	< 0.001*

*ERS *Electrical Risk Score,* SBP *Systolic blood pressure,* DBP *Diastolic blood pressure,* WBC *White blood cell count,* CRP *C-reactive protein,* GCS *Glasgow Coma Scale

*Statistically significant (*p* < 0.05)

## Discussion

Accurate and rapid risk assessment is critical in the acute management of IS. While tools such as the NIHSS and GCS are commonly used, they have limitations, especially in emergency settings where time and resources are often constrained [1, 19]. Therefore, the search for simple, objective, and easily accessible markers to assist with early diagnosis and mortality risk prediction remains an important focus in stroke research [2]. In this context, the ERS, a composite ECG-based metric, may provide additional support for early risk stratification in patients with IS, particularly when neurologic scoring or advanced imaging is not immediately available. However, our case–control design compared confirmed IS patients with healthy controls and does not fully reflect the real-world uncertainty in emergency diagnosis.

This is the first study to show that ERS, which combines six ECG parameters, demonstrated moderate discriminative performance between IS cases and controls and predicted 30-day mortality. In our study, ERS values were higher in IS patients than in healthy controls. An ERS ≥ 3 predicted IS diagnosis with 61.0% sensitivity and 77.2% specificity (PPV: 84.1%, NPV: 50.0%), whereas an ERS ≥ 4 predicted 30-day mortality with 55.2% sensitivity and 74.3% specificity (PPV: 26.7%, NPV: 90.7%).

These results suggest that ERS can support early triage and outcome prediction. Our findings align with previous studies showing that ECG abnormalities, including P-wave terminal force, QTc prolongation, and T-wave changes, are associated with worse outcomes in IS patients [20]. While ERS itself was not examined in those studies, our approach combines multiple ECG markers into a single score, which may improve clinical applicability.

Many ECG changes frequently observed in acute IS, such as QTc prolongation and ST-segment shifts, are often transient and most prominent during the acute phase [21]. Therefore, the prognostic value of isolated ECG parameters may be limited. ERS combines six variables—heart rate, QTc interval, Tp-e interval, QRS-T angle, LVH, and QRS transition—to provide a more stable and reproducible risk assessment. Each of these parameters has been independently associated with adverse cardiovascular events, including arrhythmias and sudden cardiac death [14].

The pathophysiological effects of stroke-induced autonomic dysregulation may explain the link between ERS and short-term mortality in IS. Acute infarcts involving the insular cortex have been shown to activate the sympathetic nervous system, promoting catecholamine release, myocardial injury, and arrhythmogenic electrical changes, such as QTc prolongation and repolarisation heterogeneity—all of which are captured by ERS components [22, 23]. These processes contribute to the clinical entity known as stroke–heart syndrome, where cerebral ischemia triggers adverse cardiac events, including sudden death [24]. The cumulative burden of these ECG abnormalities, reflected in a high ERS, thus serves as a surrogate marker for both neurologic insult and cardiac vulnerability.

Importantly, the relationship between stroke and cardiac dysfunction is likely bidirectional. While chronic cardiovascular conditions increase the risk of stroke, acute ischemic events can also precipitate cardiac complications through neurogenic pathways.

Our study is the first to assess their combined prognostic utility specifically in IS. It is well established that IS can induce cardiac complications through autonomic dysfunction, particularly when the right insular cortex is affected, leading to increased sympathetic activity, myocardial injury, and arrhythmia risk [3–7]. ECG markers such as QTc, Tp-e interval, and the QRS-T angle have been linked to IS severity and mortality [12, 25, 26].

However, the predictive value of these parameters individually may be compromised by confounding factors such as medications, electrolyte imbalances, or preexisting cardiac disease. While transient factors or underlying comorbidities may influence single ECG parameters, the ERS combines multiple independent variables into a composite metric, thereby reducing random variability and enhancing the robustness of clinical prognostication [15].

We found that CRP predicted 30-day mortality but was not associated with IS diagnosis, suggesting that systemic inflammation contributes to early mortality independently of cardiac stress. This aligns with previous studies linking CRP to poor outcomes in IS. Our results highlight the potential role of CRP in early mortality risk assessment in acute IS [2].

Finally, the correlation between ERS and GCS scores further supports ERS as a quick tool to estimate clinical severity at admission.

### Clinical implications

ERS may be particularly valuable in EDs where access to neuroimaging is delayed or where neurologic scoring tools are impractical. This simple ECG-derived score can help clinicians identify high-risk patients and facilitate expedited imaging, early neurology consultation, or ICU admission.

Moreover, ERS has potential utility beyond ED. Its integration into prehospital triage systems—such as ambulance-based ECG devices or mobile health platforms—could enable early risk stratification even before hospital arrival. This may help optimise resource allocation, activate IS teams sooner, and improve patient outcomes. Additionally, coupling ERS with AI-driven ECG interpretation or decision-support tools may further enhance real-time triage and early prediction of 30-day mortality.

### Future research

Prospective multicenter studies should validate ERS performance across diverse IS subtypes and ethnic populations. ERS could also be evaluated longitudinally—tracking trends post-treatment (e.g., thrombolysis) or during rehabilitation. Moreover, head-to-head comparison with NIHSS or imaging-based prognostic models will clarify ERS’s incremental value.

### Limitations

This study has several limitations. It was conducted at a single tertiary centre with a moderate sample size, which may limit generalizability. IS severity was assessed using GCS rather than NIHSS, as NIHSS was not routinely available at admission during the study period. Serial ECG recordings were not obtained, preventing evaluation of dynamic changes in ERS over time. Cardiac biomarkers such as BNP/NT-proBNP and echocardiographic data were not consistently available. Medications affecting ECG parameters (e.g., QT-prolonging agents), transient electrolyte disturbances, and possible subclinical cardiac disease may have influenced ERS values. Detailed neuroanatomical lesion localisation (e.g., hemispheric laterality or insular involvement) was not systematically analysed; therefore, the relationship between IS location and ERS could not be evaluated. Finally, the case–control design compared confirmed IS patients with healthy controls rather than patients with undifferentiated neurological presentations; thus, the reported discriminative performance may not fully reflect real-world diagnostic uncertainty.

## Conclusion

ERS, a composite ECG-based score, demonstrated moderate discriminative performance between confirmed IS cases and matched controls and independently predicted 30-day mortality. As a readily available bedside tool derived from standard 12-lead ECG, ERS may support early risk stratification in acute IS, particularly in emergency settings where rapid neurologic assessment or advanced imaging may be limited. However, ERS is not intended to replace comprehensive diagnostic evaluation but rather to complement existing clinical and imaging-based approaches.

Further large-scale, multicenter studies are warranted to validate these findings and determine the incremental value of ERS alongside established IS severity scales.

## Data Availability

The datasets generated or analysed during the current study are available from the corresponding author upon reasonable request.

## Abbreviations

AI: Artificial Intelligence
AUC: Area Under the Curve
bpm: Beats per minute
BNP: B-type Natriuretic Peptide
CAD: Coronary Artery Disease
CHF: Congestive Heart Failure
CI: Confidence Interval
CK-MB: Creatine Kinase–Myocardial Band
COPD: Chronic Obstructive Pulmonary Disease
CRP: C-reactive Protein
CT: Computed Tomography
DBP: Diastolic Blood Pressure
DM: Diabetes Mellitus
ECG: Electrocardiography
ED: Emergency Department
ERS: Electrical Risk Score
GCS: Glasgow Coma Scale
HT: Hypertension
IQR: Interquartile Range
IS: Ischemic Stroke
LVH: Left Ventricular Hypertrophy
MAP: Mean Arterial Pressure
MRI: Magnetic Resonance Imaging
NT-proBNP: N-terminal pro-B-type Natriuretic Peptide
NIHSS: National Institutes of Health Stroke Scale
OR: Odds Ratio
QTc: Corrected QT Interval
ROC: Receiver Operating Characteristic
SBP: Systolic Blood Pressure
SD: Standard Deviation
Tp-e: T peak–to–T end Interval
WBC: White Blood Cell Count
